# Celastrol Pyrazine Derivative Alleviates Silicosis Progression via Inducing ROS-Mediated Apoptosis in Activated Fibroblasts

**DOI:** 10.3390/molecules29020538

**Published:** 2024-01-22

**Authors:** Ying Bai, Chao Liang, Lu Gao, Tao Han, Fengxuan Wang, Yafeng Liu, Jiawei Zhou, Jianqiang Guo, Jing Wu, Dong Hu

**Affiliations:** 1 School of Medicine, Anhui University of Science and Technology, Huainan 232001, China; by0319_cpu@163.com (Y.B.); austlc@126.com (C.L.); austgaol@126.com (L.G.); austhant@126.com (T.H.); fengxuan_1620@163.com (F.W.); austliuyf@126.com (Y.L.); austzhoujw@126.com (J.Z.); austguojq@126.com (J.G.); 2 Anhui Occupational Health and Safety Engineering Laboratory, Huainan 232001, China; 3 Key Laboratory of Industrial Dust Deep Reduction and Occupational Health and Safety of Anhui Higher Education Institute, Huainan 232001, China; 4 Key Laboratory of Industrial Dust Prevention and Control and Occupational Safety and Health Ministry of Education, Anhui University of Science and Technology, Huainan 232001, China

**Keywords:** silicosis, pulmonary fibrosis, celastrol derivative, fibroblast, apoptosis

## Abstract

Silicosis is a complex occupational disease without recognized effective treatment. Celastrol, a natural product, has shown antioxidant, anti-inflammatory, and anti-fibrotic activities, but the narrow therapeutic window and high toxicity severely limit its clinical application. Through structural optimization, we have identified a highly efficient and low-toxicity celastrol derivative, **CEL-07**. In this study, we systematically investigated the therapeutic potential and underlying mechanisms of **CEL-07** in silicosis fibrosis. By constructing a silicosis mouse model and analyzing with HE, Masson, Sirius Red, and immunohistochemical staining, **CEL-07** significantly prevented the progress of inflammation and fibrosis, and it effectively improved the lung respiratory function of silicosis mice. Additionally, **CEL-07** markedly suppressed the expression of inflammatory factors (IL-6, IL-1α, TNF-α, and TNF-β) and fibrotic factors (α-SMA, collagen I, and collagen III), and promoted apoptosis of fibroblasts by increasing ROS accumulation. Moreover, bioinformatics analysis combined with experimental validation revealed that **CEL-07** inhibited the pathways associated with inflammation (PI3K-AKT and JAK2-STAT3) and the expression of apoptosis-related proteins. Overall, these results suggest that **CEL-07** may serve as a potential candidate for the treatment of silicosis.

## 1. Introduction

Silicosis is a progressive and irreversible disease, in which residual crystalline silica (CS) causes persistent inflammation, tissue damage, and fibrosis, ultimately leading to respiratory failure and death [1,2,3,4]. Currently available therapies can only slow down the progression of the disease but cannot reverse the progress of silicosis. Western medicine mainly uses bronchial lavage, stem cell therapy, lung transplantation, and interventions with drugs such as polyvinylpyridine-oxide, piperaquine phosphate, glucocorticoid, and infliximab [5,6], but the therapeutic effects are limited and side effects are frequent. Traditional Chinese medicine mainly uses trehalose [7], tetrandrine [8], and atractylidene-1 [9] as supplementary treatments, which can alleviate the clinical symptoms of silicosis. However, they are mostly used as adjunctive therapies. In addition, two drugs approved for idiopathic pulmonary fibrosis (IPF), pirfenidone [10] and nintedanib [11], have shown improvement in silicosis in preclinical studies. The proinflammatory factor inhibitor Anakinra has also been reported to improve the respiratory function of silicosis patients through subcutaneous injection for 6 months [12]. At present, the existing therapeutic strategies have not made breakthrough progress and there is an urgent need to develop new drugs and effective means to treat silicosis.

**Figure 1:**
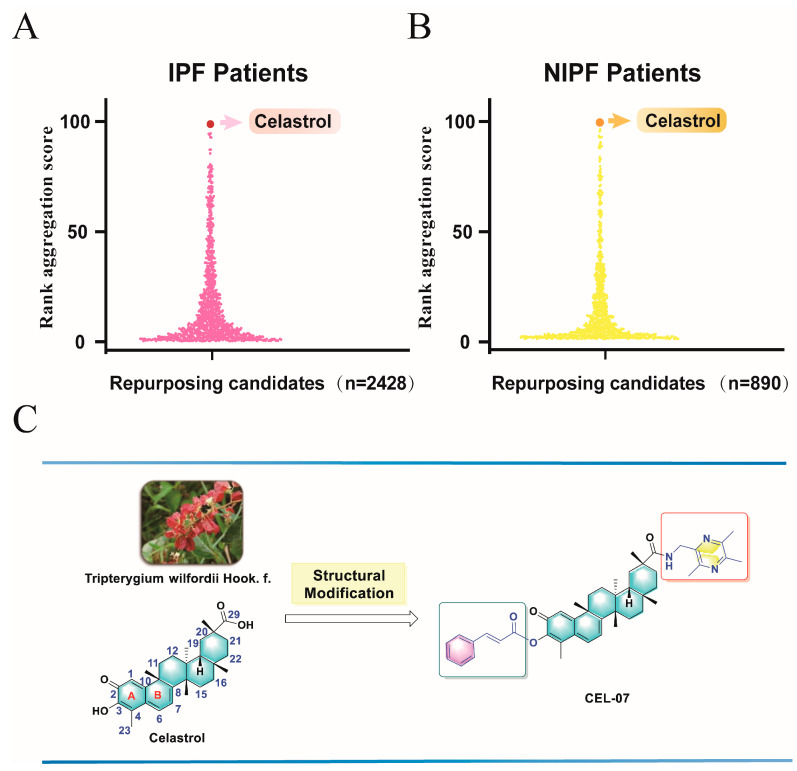
The process of screening and discovering compound **CEL-07**. (**A**) The drugs available for IPF treatment screened by cMap. (**B**) The drugs available for non-IPF treatment screened by cMap. (**C**) Compound **CEL-07** was obtained by structural optimization of celastrol.

In our previous studies, we identified differentially expressed genes between patients with idiopathic pulmonary fibrosis (IPF) and non-idiopathic pulmonary fibrosis (Non-IPF) through analysis of gene sequencing results, and imported significantly upregulated and downregulated genes into the cMap database for potential therapeutic drug screening [13,14,15]. The screened drugs were sorted according to their scores, and we were surprised to find that regardless of whether it was IPF or non-IPF, celastrol was the most promising small-molecule drug, worthy of further research (Figure 1).

Celastrol (CSL) is a natural pentacyclic triterpene of the Celastraceae family and one of the active ingredients of the Tripterygium wilfordii Hook.f. plant. It has various pharmacological activities, such as anti-rheumatoid arthritis, anti-tumor, antioxidant, neuroprotection, and blood-glucose-lowering effects [16,17,18,19]. In 2007, it was listed as one of the five natural products most likely to be developed into drugs by the journal Cell [20]. In 2015, Umut Ozcan’s team found that CSL can significantly reduce the weight of obese mice by improving leptin sensitivity and reducing food intake, becoming a highly potential new weight-loss drug [21]. Although CSL has a wide range of pharmacological activities, it has substantial toxicity, a narrow therapeutic window, and a median lethal dose of approximately 20.5 mg/kg [22]. Reports have confirmed that CSL has hepatorenal toxicity, hematotoxicity, reproductive toxicity, and embryonic development toxicity during clinical application [23,24]. Therefore, the potential toxic effects of CSL are key factors limiting its development into a drug, and how to reduce its toxicity and enhance its effectiveness is an important scientific problem that needs to be addressed. In addition, the poor water solubility and low oral bioavailability of CSL also limit its clinical application. Therefore, the structural modification and optimization of CSL have become a hot topic of attention both domestically and internationally [25,26].

In our preliminary research, we optimized the structure of CSL and discovered compound **CEL-07**, which significantly improved water solubility and potential toxicity compared to celastrol (Figure 1) [27,28]. However, the role and mechanism of **CEL-07** in the treatment of silicosis remains unclear. In this study, we first established a mouse model of silicosis and studied the in vivo potential of compound **CEL-07** to improve inflammation and fibrosis in silicosis. We also explored the mechanisms of **CEL-07** in treating silicosis at the cellular level. This study aims to identify a potential candidate compound for the treatment of silicosis.

## 2. Results

### 2.1. **CEL-07** Inhibits Pulmonary Pathological Damage in CS-Induced Silicosis Mice

**Figure 2:**
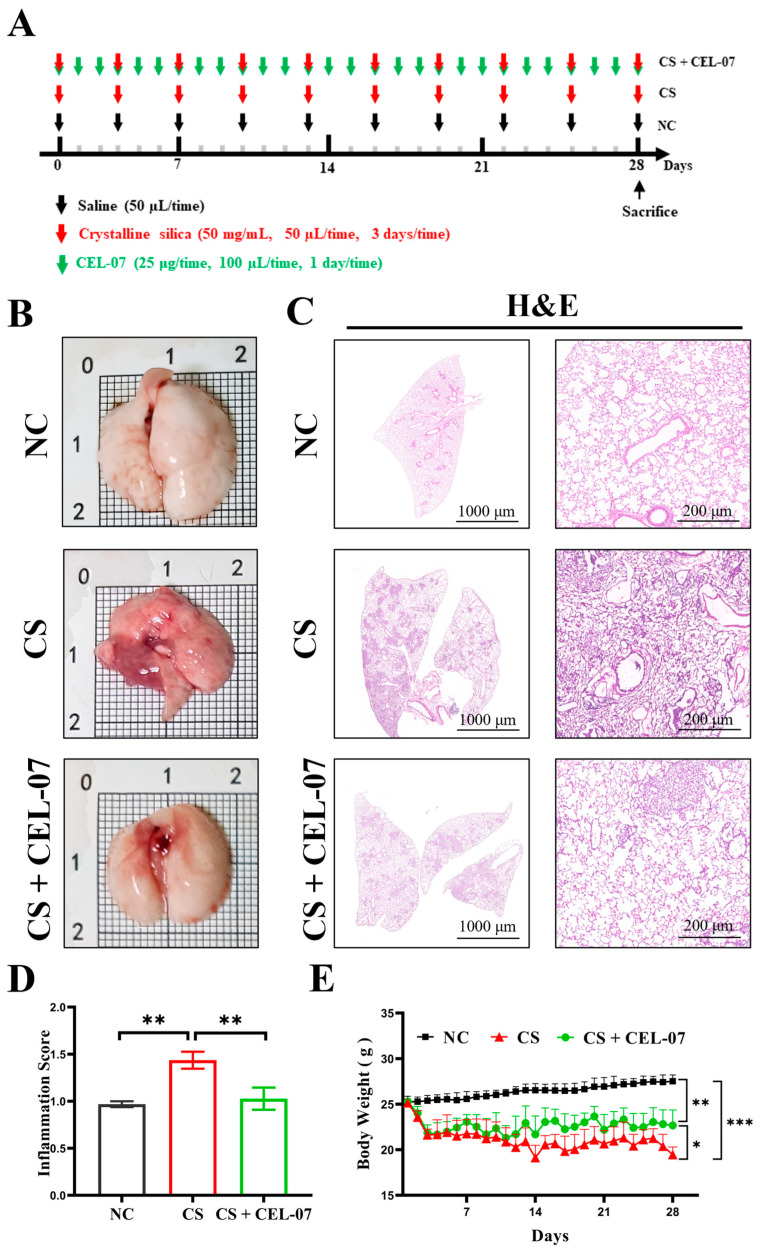
**CEL-07** alleviates lung injury induced by CS in mice. (**A**) Schematic representation of CS-induced lung injury mouse model. CS suspension (50 mg/mL, 50 μL) was instilled into the nasal cavity every three days, and **CEL-07** (25 μg, 100 μL) was intraperitoneally injected daily. (**B**) Dissection of mouse lung tissues. (**C**) Histopathological analysis of lung tissue inflammation using HE staining. (**D**) Scoring of lung tissue inflammation. (**E**) Body weight changes of mice. NC: Negative control group; CS: CS-induced group; CS + **CEL-07**: **CEL-07** treatment group. (*) *p* < 0.05. (**) *p* < 0.01. (***) *p* < 0.001. Error bars represent mean ± SD.

To investigate the therapeutic effect of **CEL-07** on CS-induced lung fibrosis in mice, we established a CS-induced silicosis mouse model. The model was developed by intranasal administration of CS suspension (50 mg/mL, 50 μL). The mice were divided into three groups: control group (NC), model group (CS), and treatment group (CS + **CEL-07**) (Figure 2A). After 28 days of modeling, we assessed the lung tissue morphology. The results showed that the CS group exhibited increased lung tissue volume and a dark red appearance, whereas the CS + **CEL-07** group showed no obvious changes in lung tissue volume and a smooth surface (Figure 2B). HE staining revealed that the NC group had a normal alveolar wall structure without inflammatory cell infiltration, while the CS group exhibited significant lung inflammation and infiltration of inflammatory cells. However, treatment with **CEL-07** reduced inflammation and alleviated lung tissue damage (Figure 2C). Histological scoring of lung tissues showed a significant decrease in inflammation in the CS + **CEL-07** group compared to the CS group (Figure 2D). Additionally, the CS group showed a decrease in body weight, whereas the CS + **CEL-07** group had a stable body weight (Figure 2E). These results indicate that CS induces lung tissue inflammation and pathological damage, but **CEL-07** treatment can effectively alleviate these effects.

### 2.2. **CEL-07** Suppresses CS-Induced Lung Fibrosis

**Figure 3:**
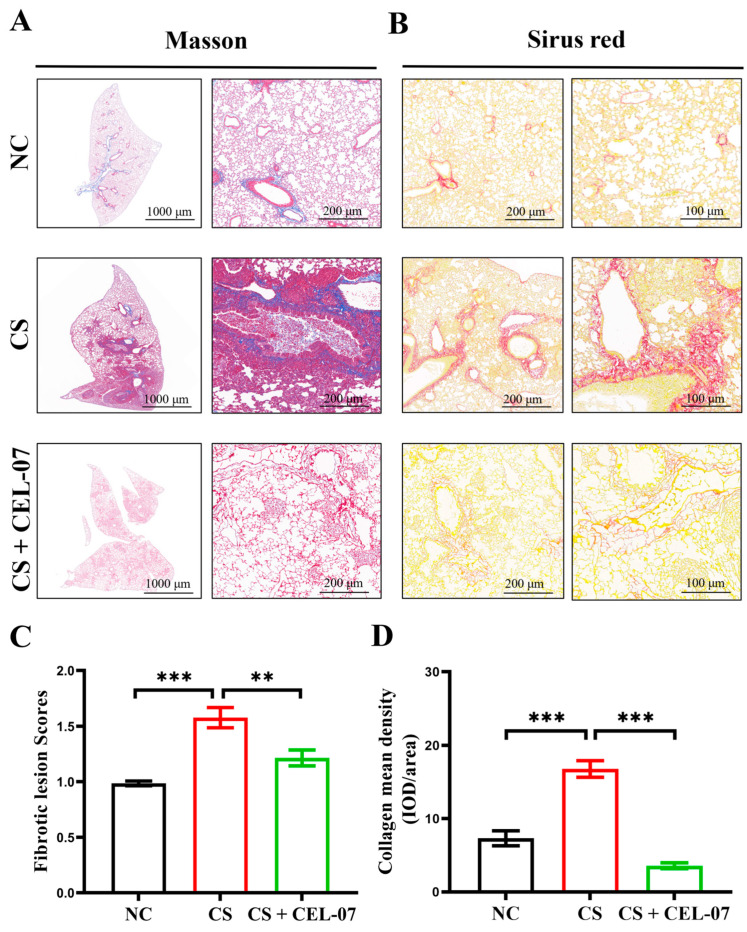
**CEL-07** alleviates lung fibrosis in CS-induced mice. (**A**) Analysis of fibrosis severity in mouse lung tissues using Masson staining. (**B**) Analysis of collagen fiber deposition in mouse lung tissues using Sirius Red staining. (**C**) Quantification of the fibrotic area in Masson staining. (**D**) Quantification of collagen fiber area in Sirius Red staining. NC: Negative control group; CS: CS-induced group; CS + **CEL-07**: **CEL-07** treatment group. (**) *p* < 0.01. (***) *p* < 0.001. Error bars represent mean ± SD.

The inhalation of silica particles into the lungs triggers a cascade of events, as these particles are not readily engulfed by macrophages. Consequently, the activated macrophages release various cytokines to stimulate fibroblast activation and proliferation. Simultaneously, the accumulation of extracellular matrix leads to progressive pulmonary fibrosis. To determine whether **CEL-07** can improve CS-induced lung fibrosis, we performed Masson staining and Sirius Red staining to assess fibrosis in lung tissues. The results showed that the CS group had significant fibrosis with increased collagen fiber deposition, while the CS + **CEL-07** group exhibited reduced fibrosis with decreased collagen deposition (Figure 3A,B). The assessment of fibrotic area and collagen fiber content confirmed that **CEL-07** significantly reduced fibrosis in lung tissues (Figure 3C,D). These results indicate that **CEL-07** can suppress CS-induced lung fibrosis by inhibiting the production of collagen fibers.

### 2.3. **CEL-07** Reduces Excessive Extracellular Matrix Deposition in Silicosis Mice

**Figure 4:**
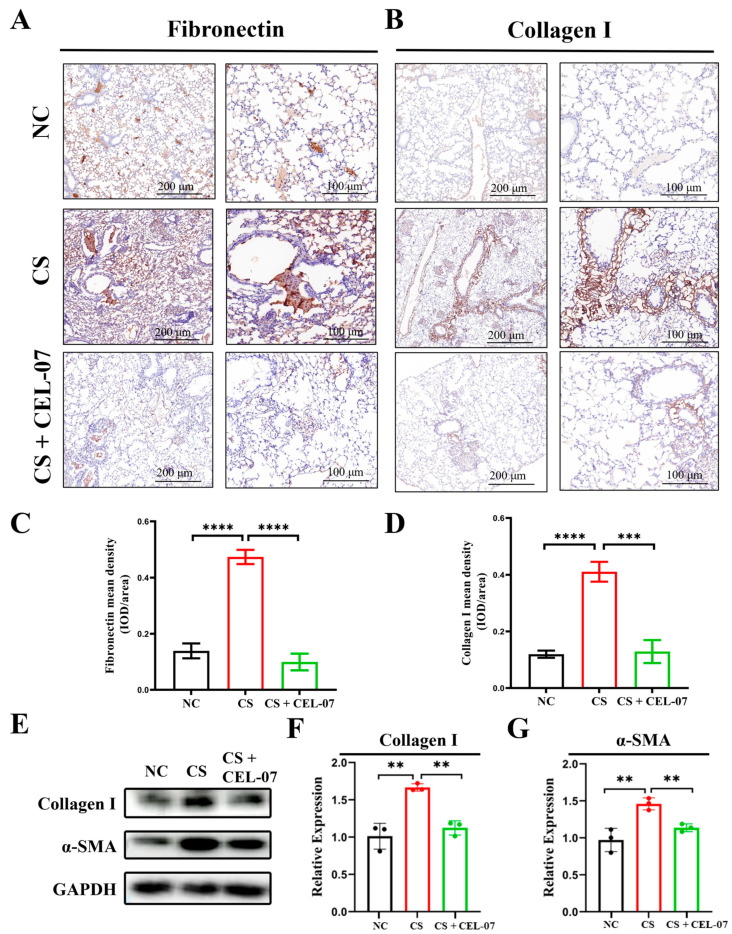
**CEL-07** reduces excessive extracellular matrix deposition in silicosis mice. (**A**) Immunohistochemical analysis of fibronectin expression in different groups of mouse lung tissues. (**B**) Immunohistochemical analysis of collagen I expression in different groups of mouse lung tissues. (**C**) Quantification of fibronectin immunostaining intensity. (**D**) Quantification of collagen I immunostaining intensity. (**E**–**G**) Western blot analysis of protein expression of collagen I and α-SMA and quantification of protein expression. NC: Negative control group; CS: CS-induced group; CS + **CEL-07**: **CEL-07** treatment group. (**) *p* < 0.01. (***) *p* < 0.001. (****) *p* < 0.0001. Error bars represent mean ± SD.

The excessive deposition of extracellular matrix (ECM) proteins, such as fibronectin and collagen, plays a crucial role in the development of lung fibrosis. The accumulation of ECM disrupts normal lung architecture and impairs lung function. Understanding and targeting the ECM deposition process may offer potential therapeutic strategies for preventing or reversing the fibrotic tissue deposition in silicosis, thereby improving lung function. To evaluate the effect of **CEL-07** on ECM deposition in CS-induced lung fibrosis, immunohistochemical staining was performed to assess the expression of fibronectin and collagen I in lung tissues. The results showed that the expression of fibronectin and collagen I was significantly increased in the CS group compared to the NC group, indicating the deposition of ECM proteins in lung tissues. Treatment with **CEL-07** significantly decreased the expression of fibronectin and collagen I in lung tissues (Figure 4). These results suggest that **CEL-07** can effectively inhibit excessive ECM deposition in CS-induced lung fibrosis.

### 2.4. **CEL-07** Improves Lung Respiratory Function of Silicosis Mice

**Figure 5:**
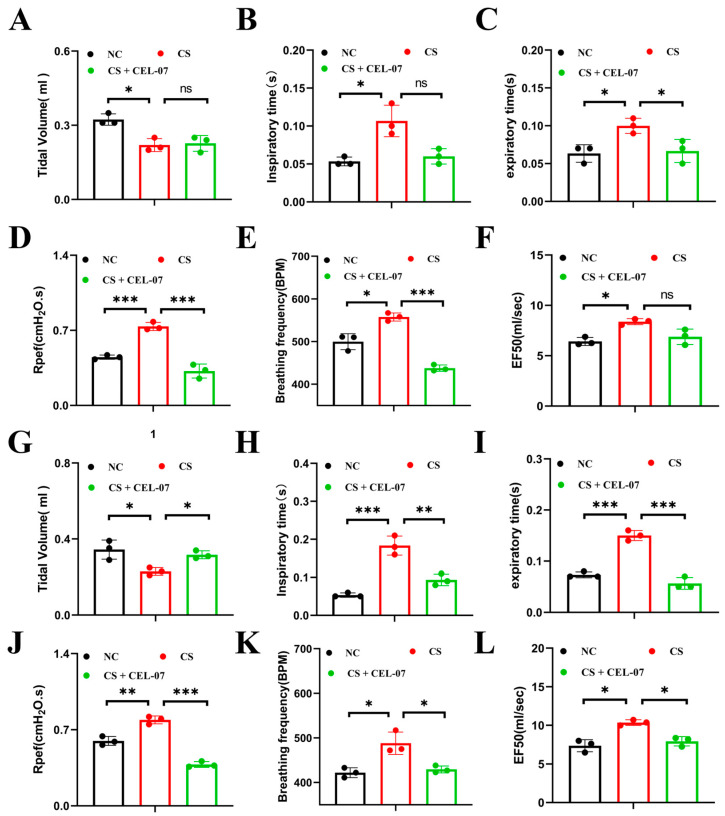
**CEL-07** improves respiratory function of silicosis mice. (**A**–**F**) Noninvasive plethysmography was used to measure respiratory function parameters, including breathing time, respiratory rate, tidal volume, specific airway resistance, and median expiratory flow rate, on day 14 in different groups of mice. (**G**–**L**) Noninvasive plethysmography was used to measure respiratory function parameters on day 28 in different groups of mice. NC: Negative control group; CS: CS-induced group; CS + **CEL-07**: **CEL-07** treatment group. (ns) *p* > 0.05. (*) *p* < 0.05. (**) *p* < 0.01. (***) *p* < 0.001. Error bars represent mean ± SD.

As the silicosis progresses, the lung’s ability to efficiently exchange oxygen and carbon dioxide is compromised, resulting in impaired pulmonary respiratory function. This manifests as decreased lung capacity, reduced oxygen uptake, and difficulty in breathing. Monitoring and improving pulmonary respiratory function are essential in managing and treating silicosis, as it can greatly impact overall respiratory health and quality of life for individuals affected by the disease. The whole-body plethysmography was used to evaluate lung function in mice. The parameters measured included respiratory time, respiratory rate, tidal volume, specific airway resistance, and median expiratory flow rate. The results showed that the CS group exhibited a significant decrease in tidal volume and an increase in respiratory rate, airway resistance, and median expiratory flow rate, indicating impaired lung function (Figure 5). Treatment with **CEL-07** improved these lung function parameters, suggesting that **CEL-07** can effectively improve lung respiratory function of silicosis mice.

### 2.5. **CEL-07** Suppresses the Expression of Inflammatory and Fibrotic Factors in Silicosis Mice

**Figure 6:**
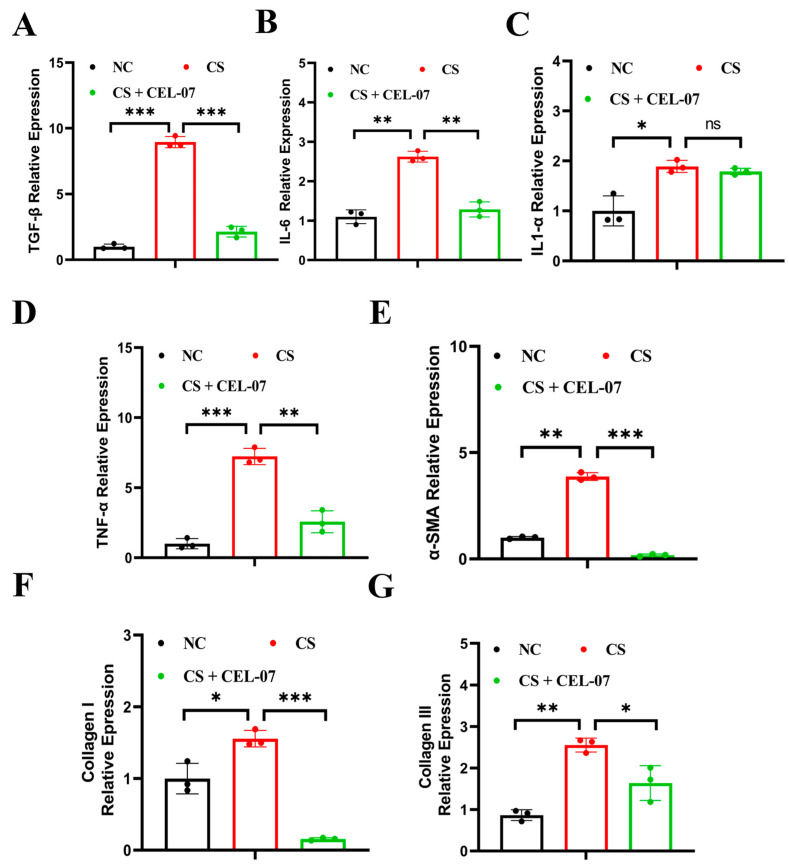
**CEL-07** suppresses the expression of inflammatory and fibrotic factors in silicosis mice. (**A**–**G**) Real-time PCR analysis of inflammatory factors (TNF-α, TGF-β, IL-6, IL-1α, IL-1β) and fibrotic factors (α-SMA, collagen I, collagen III) in mouse lung tissues. NC: Negative control group; CS: CS-induced group; CS + **CEL-07**: **CEL-07** treatment group. (ns) *p* > 0.05. (*) *p* < 0.05. (**) *p* < 0.01. (***) *p* < 0.001. Error bars represent mean ± SD.

Inflammation and fibrosis are hallmarks of lung fibrosis, and the release of various cytokines plays a crucial role in these processes. To investigate the effect of **CEL-07** on the expression of inflammatory and fibrotic factors in lung tissues, we performed RT-qPCR analysis. The results showed that the expression of inflammatory cytokines (IL-6, IL-1α, TNF-α, and TNF-β) was significantly increased in the CS group compared to the NC group. Treatment with **CEL-07** reduced the expression of these inflammatory factors (Figure 6A–D). Additionally, the expression of fibrosis-related factors (α-SMA, collagen I, and collagen III) was increased in the CS group, and **CEL-07** treatment significantly decreased their expression (Figure 6E–G). These results indicate that **CEL-07** can suppress the expression of inflammatory and fibrotic factors in the lung tissues of silicosis mice.

### 2.6. **CEL-07** Inhibits the Expression of Inflammatory and Fibrotic Factors In Vitro

**Figure 7:**
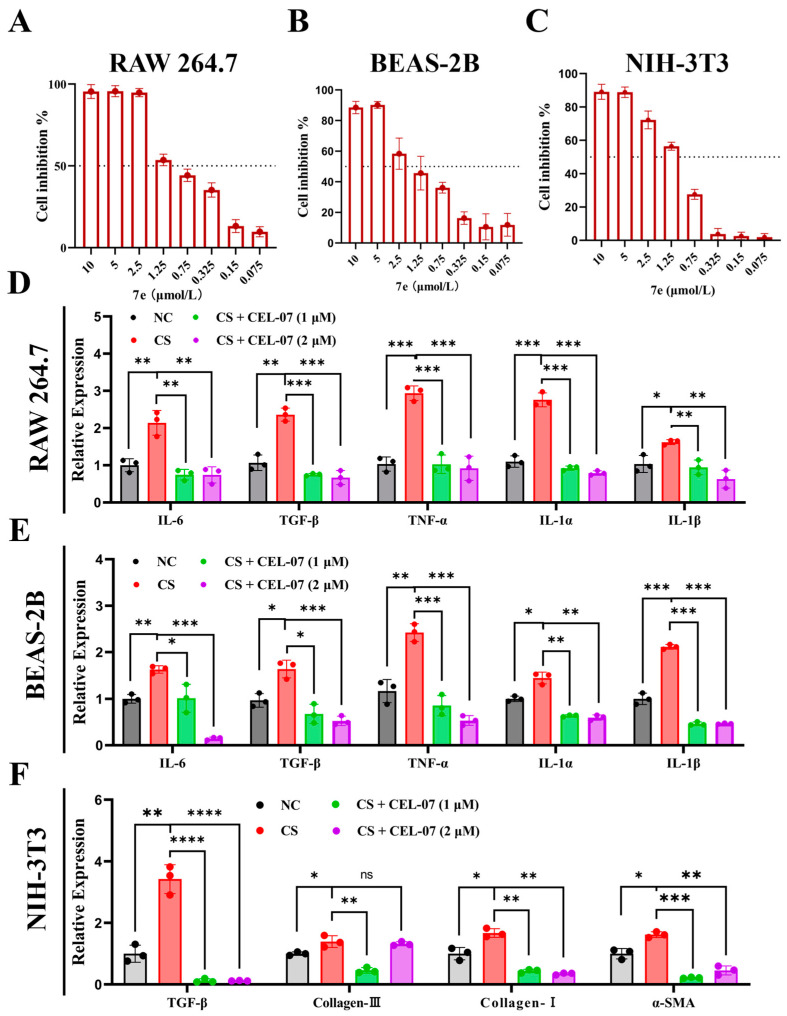
**CEL-07** inhibits the expression of inflammatory and fibrotic factors in vitro. (**A**–**C**) Cell viability of different concentrations of **CEL-07** in RAW264.7, BEAS-2B, and NIH-3T3 cells. (**D**–**F**) RT-qPCR analysis of TNF-α, TGF-β, IL-6, IL-1β, IL-1α, α-SMA, collagen I, and collagen III gene expression in cells stimulated with CS. NC: Negative control group; CS: CS-induced group; CS + **CEL-07**: **CEL-07** treatment group. (ns) *p* > 0.05. (*) *p* < 0.05. (**) *p* < 0.01. (***) *p* < 0.001. (****) *p* < 0.0001. Error bars represent mean ± SD.

To further investigate the effect of **CEL-07** on the expression of inflammatory and fibrotic factors, we constructed CS-induced macrophages (RAW264.7), epithelial cells (BEAS-2B), and fibroblasts (NIH-3T3) and assessed the expression of inflammatory and fibrotic factors. To determine the appropriate dosing concentration of **CEL-07** in the three cell lines, we performed a declining-down cell activity assay using the CCK-8 assay, which ultimately determined the dosing concentration to be 1–2 μM (Figure 7A–C). The results of RT-qPCR showed that **CEL-07** significantly inhibited the expression of inflammatory factors (IL-1β, IL-1α, IL-6, TNF-α, and TGF-β) in CS-activated RAW264.7 and BEAS-2B (Figure 7D,E). Moreover, the culture medium from CS-stimulated macrophages promoted the expression of fibrotic factors (TGF-β, α-SMA, collagen I, and collagen III) in fibroblasts, while treatment with **CEL-07** attenuated their expression (Figure 7F). These results indicate that **CEL-07** can suppress the expression of inflammatory and fibrotic factors in vitro.

### 2.7. **CEL-07** Promotes CS-Induced Fibroblast Apoptosis

**Figure 8:**
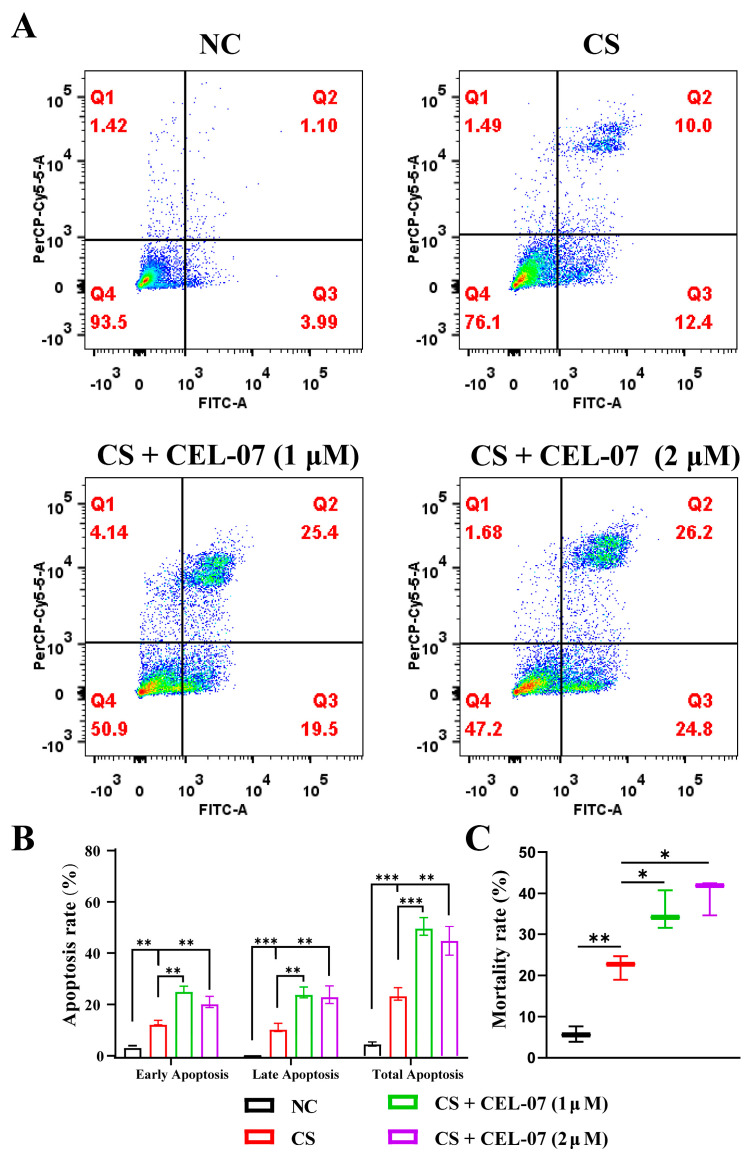
**CEL-07** promotes apoptosis of CS-induced fibroblasts. (**A**) Apoptosis of fibroblasts induced by **CEL-07** (2/4 μM) treatment for 24 h. (**B**) Histogram showing the percentage of apoptotic cells. (**C**) The number of cell deaths analyzed by trypan blue staining. NC: Negative control group; CS: CS-induced group; CS + **CEL-07**: **CEL-07** treatment group. (*) *p* < 0.05. (**) *p* < 0.01. (***) *p* < 0.001. Error bars represent mean ± SD.

In pulmonary fibrosis, excessive proliferation and activation of fibroblasts result in the accumulation of fibrotic tissue in the lungs. However, the balance between fibroblast proliferation and apoptosis is disrupted, leading to a relative deficiency of fibroblast apoptosis. This impaired apoptosis contributes to the persistence and progression of fibrosis by allowing fibroblasts to continuously produce excessive extracellular matrix. Promoting fibroblast apoptosis may be a potential therapeutic strategy to mitigate pulmonary fibrosis and reduce fibrotic tissue deposition in the lungs [29]. To investigate whether **CEL-07** can promote fibroblast apoptosis, we performed flow cytometry analysis and trypan blue staining in NIH-3T3 cells. The results showed that CS treatment induced fibroblast apoptosis, and treatment with **CEL-07** increased the percentage of early and late apoptotic cells in a concentration-dependent manner (Figure 8A–C). These findings suggest that **CEL-07** can promote CS-induced fibroblast apoptosis, thereby inhibiting fibrosis.

### 2.8. **CEL-07** Promotes Fibroblast Apoptosis by Increasing ROS Generation

**Figure 9:**
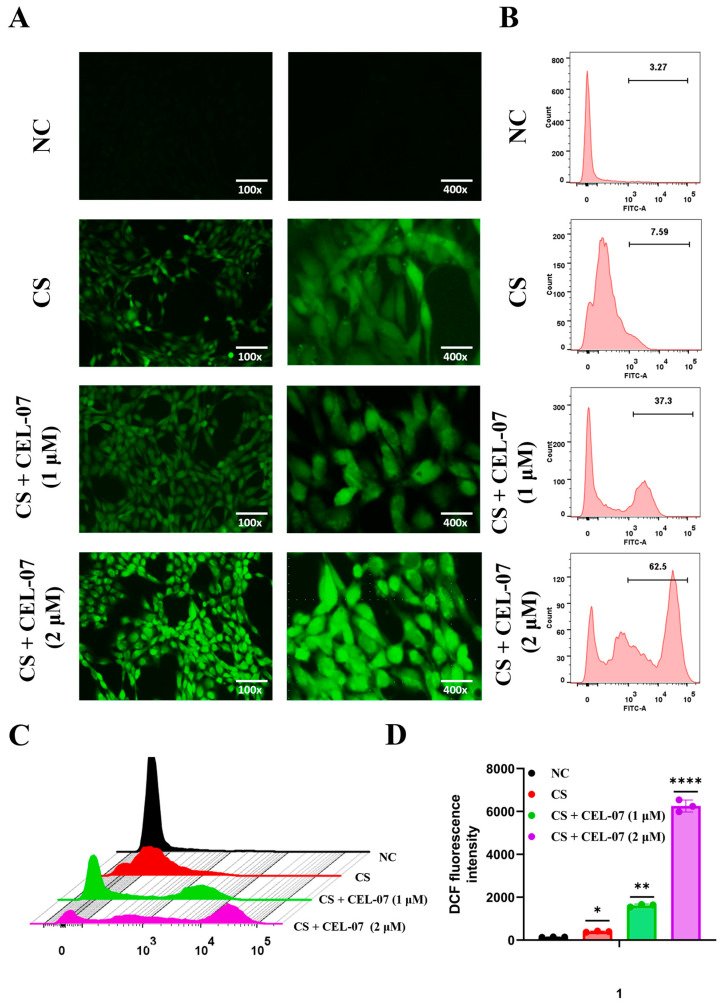
**CEL-07** promotes fibroblast apoptosis by increasing ROS generation. (**A**) Fluorescence microscopy analysis of ROS levels in NIH-3T3 cells treated with **CEL-07**. (**B**) Flow cytometry analysis of ROS levels in NIH-3T3 cells treated with **CEL-07**. (**C**) Comparison of flow cytometry results. (**D**) Histogram of mean fluorescence intensity of ROS. NC: Negative control group; CS: CS-induced group; CS + **CEL-07**: **CEL-07** treatment group. (*) *p* < 0.05. (**) *p* < 0.01. (****) *p* < 0.0001. Error bars represent mean ± SD.

Reactive oxygen species (ROS) play a crucial role in the regulation of cellular apoptosis. When cells are under stress or exposed to certain stimuli, an increase in ROS production can occur. Elevated levels of ROS can result in oxidative damage to cellular components, such as DNA, proteins, and lipids. This oxidative stress can trigger signaling pathways that lead to apoptosis. ROS can directly cause DNA damage and activate downstream apoptotic cascades involving caspases and mitochondrial dysfunction [30]. To investigate whether **CEL-07** promotes fibroblast apoptosis by increasing ROS generation, we analyzed ROS levels in fibroblasts using immunofluorescence and flow cytometry. The results showed that treatment with **CEL-07** significantly increased the generation of ROS in fibroblasts in a concentration-dependent manner (Figure 9A–D). These results suggest that **CEL-07** can enhance ROS generation in fibroblasts, thereby promoting fibroblast apoptosis.

### 2.9. Bioinformatics Analysis of the Pathogenesis and Potential Pathways of Silicosis

**Figure 10:**
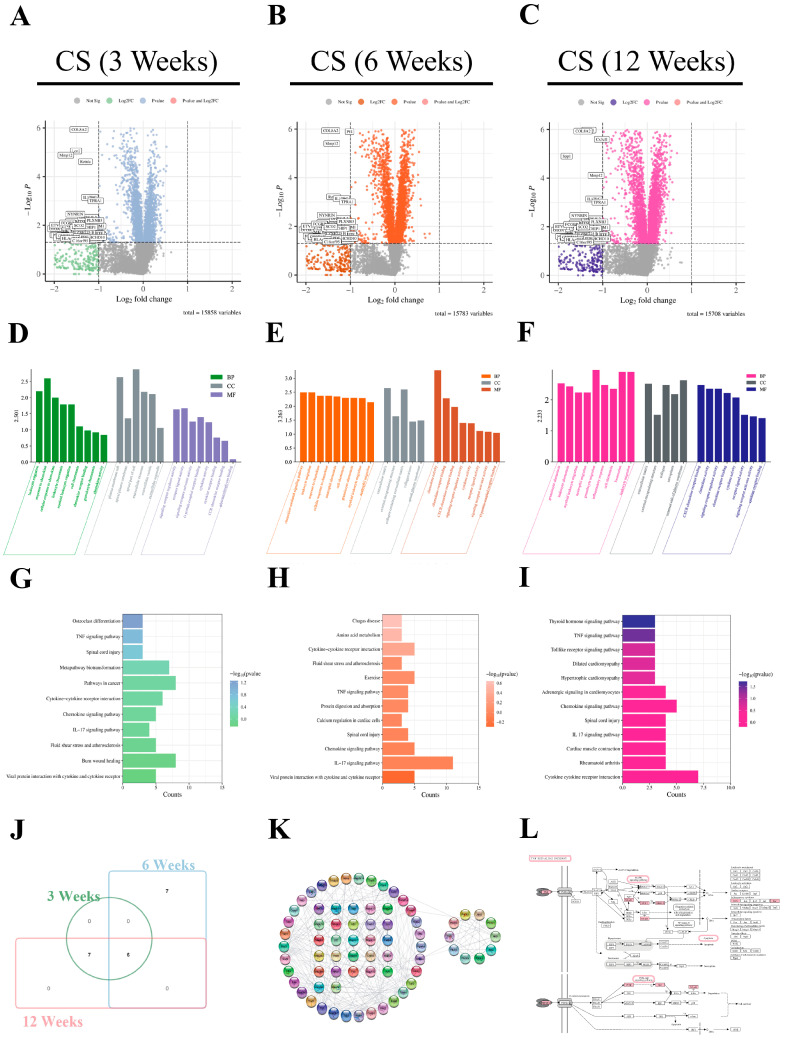
Bioinformatics analysis of the pathogenesis and potential pathways of silicosis. (**A**–**C**) Volcano plots showing differentially expressed genes in rats exposed to CS for 3, 6, and 12 weeks compared to the normal group. (**D**–**F**) Gene ontology analysis of differentially expressed genes in rats exposed to CS for 3, 6, and 12 weeks. (**G**–**I**) KEGG pathway analysis of differentially expressed genes in rats exposed to CS for 3, 6, and 12 weeks. (**J**) Venn diagram showing the enriched pathways. (**K**) Protein–protein interaction network of TNF signaling pathway. (**L**) The adherens junction pathway map of the TNF signaling pathway. Pink rectangles represent target genes involved in inflammation resistance.

To explore the potential key pathways involved in silicosis fibrosis and elucidate the mechanism of **CEL-07** in treating silicosis, we performed bioinformatics analysis. Differentially expressed genes (DEGs) were identified using the GSE49144 dataset (Figure 10A–C), and GO analysis revealed that these DEGs were mainly associated with T cell migration, chemokine activity, and inflammatory response (Figure 10D–F). KEGG analysis identified several signaling pathways, including the IL-17 signaling pathway, TNF signaling pathway, and cytokine–cytokine receptor interaction, which are closely related to inflammation and fibrosis (Figure 10G–J). The adherens junction pathway map and PPI network were constructed, and key genes such as MAPK3, IL-6, TNF-α, PI3K, and AKT were identified (Figure 10K,L). These findings provide insight into the potential key pathways and genes involved in silicosis.

### 2.10. Pathway Analysis of CEL-07 in Improving Silicosis

**Figure 11:**
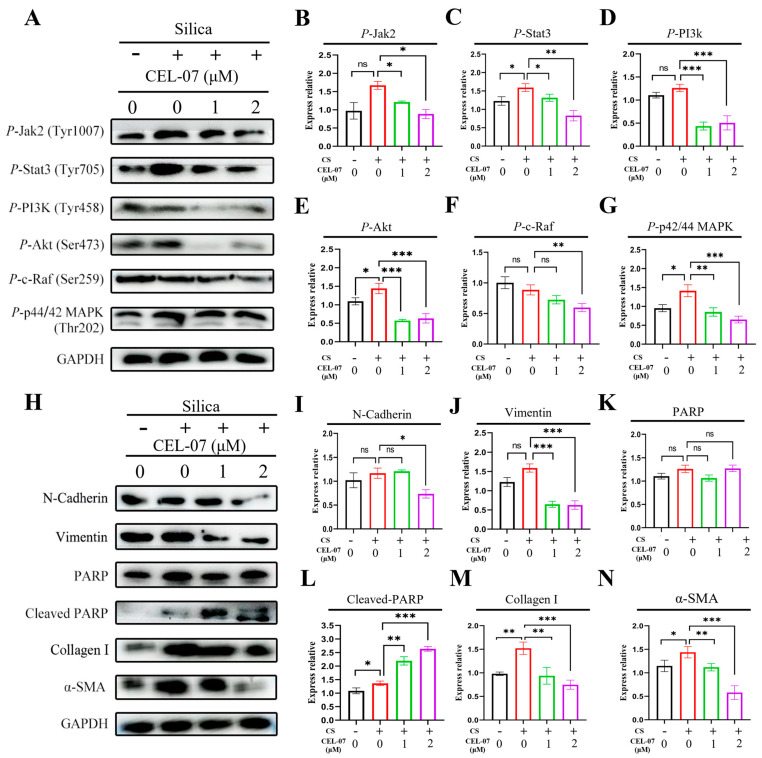
Pathway analysis of **CEL-07** in improving silicosis. (**A**–**G**) Western blot analysis of phosphorylated proteins in the JAK2-STAT3, PI3K-AKT, and MAPK signaling pathways and quantification of protein expression. (**H**–**N**) Western blot analysis of protein expression of N-Cadherin, vimentin, PARP, collagen I, α-SMA, and cleaved PARP and quantification of protein expression. NC: Negative control group; CS: CS-induced group; CS + **CEL-07**: **CEL-07** treatment group. (ns) *p* > 0.05. (*) *p* < 0.05. (**) *p* < 0.01. (***) *p* < 0.001. Error bars represent mean ± SD.

To explore whether **CEL-07** regulates the predicted key signaling pathways, Western blot assay was used to examine the phosphorylation of the MAPK, PI3K-AKT and JAK2-STAT3 signaling pathways in NIH-3T3 cells. The results showed that **CEL-07** inhibited the phosphorylation of the three signaling pathways to varying degrees (Figure 11A–G). The PI3K-AKT and JAK2-STAT3 pathways are closely related to the secretion of inflammatory factors. Activated PI3K can lead to the phosphorylation and activation of AKT, which in turn promotes the expression and release of inflammatory cytokines. Meanwhile, activation of JAK2 leads to the phosphorylation and activation of STAT3, which promotes the expression of proinflammatory cytokines and other genes involved in inflammatory responses. Therefore, we speculated that **CEL-07** may decrease inflammatory infiltration in lung tissues by suppressing PI3K-AKT and JAK2-STAT3 pathways. The MAPK signaling pathway plays a key role in cellular stress responses. When cells are stimulated, the MAPK pathway is activated, which triggers a series of biological reactions that include the production of ROS. ROS are cytotoxic and can cause DNA damage, protein oxidation, and cell structural disruption. In addition, the MAPK pathway promotes apoptosis by regulating the expression of apoptosis-related genes, such as BCL-2 family proteins and caspases. Thus, the rapid accumulation of ROS and increased apoptosis of fibroblasts treated with **CEL-07** in our research might be closely related to the activation of the MAPK pathway [31,32,33]. Furthermore, we analyzed the expression of fibrosis-related and apoptosis-related proteins. The results demonstrated that **CEL-07** markedly decreased the expression of N-cadherin and vimentin, two markers of mesenchymal cells, and the expression of collagen I and α-SMA, two markers of fibrosis cells. Furthermore, the expression of cleaved PARP, a marker of the apoptotic pathway associated with the inhibition of the DNA damage repair process, was increased in a dose-dependent manner of **CEL-07** (Figure 11H–L). This also reflected that **CEL-07** could induce apoptosis in fibroblast cells.

## 3. Discussion

Silicosis is a typical occupational disease, in which silica particles are ingested by macrophages in the lung tissue [34,35]. The release of various cytokines by macrophages causes inflammation, fibroblast proliferation, and collagen deposition in the lung tissue, leading to fibrosis [36,37,38]. Currently, there is a lack of effective drugs for the treatment of silicosis. In our previous studies, we identified celastrol as a potential therapeutic drug for silicosis, but its high toxicity and narrow therapeutic window severely limit its clinical application. Through structural optimization of celastrol, we discovered a highly effective and low-toxicity derivative, **CEL-07**.

In this study, we constructed a CS-induced early-stage silicosis mouse model to evaluate the potential therapeutic effects of **CEL-07**. The results showed significant improvements in inflammation, fibrosis, and lung respiratory function in the silicosis mice treated with intraperitoneal injection of **CEL-07**. Consistent with our findings, increasing evidence has demonstrated the ability of celastrol, the parent compound of **CEL-07**, to improve fibrosis in other organs such as the liver and kidney. Furthermore, we found that **CEL-07** significantly inhibited the expression of collagen protein and fibronectin, indirectly confirming its good effect in inhibiting fibroblast proliferation. Additionally, **CEL-07** demonstrated a favorable effect in suppressing the release of inflammatory factors by macrophages and epithelial cells, while also significantly reducing the secretion of fibrotic factors by fibroblasts. Further apoptosis experiments confirmed that **CEL-07** can promote ROS-mediated apoptosis of fibroblasts in a concentration-dependent manner. Oxidative stress is a cellular stress state induced by the disruption of the intracellular redox balance, leading to an excessive production of ROS, which include superoxide anions, hydrogen peroxide, and hydroxyl radicals. Oxidative stress can be triggered by various factors, such as increased free radical generation, damaged antioxidant defense systems, and abnormal mitochondrial function. Oxidative stress activates inflammation, which in turn can generate more ROS. These ROS can activate various signaling pathways and transcription factors, such as nuclear factor κB (NF-κB) and a series of mitogen-activated protein kinases (MAPK). The activation of these signaling pathways and transcription factors promotes the generation and release of inflammatory cytokines, forming a self-perpetuating cycle of inflammation and oxidative stress [39]. However, in some cases, inflammation-induced cell apoptosis can serve as a negative feedback mechanism to control excessive inflammation. Apoptosis is a controlled form of cell death distinct from necrosis. Apoptotic cells release anti-inflammatory signals and prevent the spread of inflammation [40]. For example, apoptotic cells secrete TGF-β to inhibit inflammation and promote repair processes. Additionally, anti-inflammatory proteases released by apoptotic cells activate receptors (PAR) that suppress the release of inflammatory cytokines. Therefore, inflammation-induced cell apoptosis plays a crucial role in regulating inflammation and maintaining tissue homeostasis.

To investigate the underlying mechanism, we analyzed the transcriptomic data of rat lung tissues at different stages and found significant correlations between the progression of silicosis and the MAPK, JAK-STAT, and PI3K-AKT pathways. Therefore, we selectively tested the phosphorylation inhibition of the three pathways by **CEL-07**, and the results showed that **CEL-07** significantly inhibited the activation of the JAK-STAT and PI3K-AKT signaling pathways. Both the JAK-STAT and PI3K-AKT pathways play significant roles in the occurrence and development of silicosis. An activated JAK-STAT pathway participates in the regulation of inflammation and fibrosis. It promotes the expression of proinflammatory cytokines and chemokines, leading to the recruitment and activation of immune cells. Furthermore, the JAK-STAT pathway is involved in the fibrotic process by stimulating the production of extracellular matrix components, such as collagen [41,42]. On the other hand, the PI3K-AKT pathway is crucial in regulating cell survival and growth. Activation of this pathway promotes cell proliferation and inhibits apoptosis, contributing to the excessive activation and proliferation of fibroblasts in silicosis. Moreover, the PI3K-AKT pathway interacts with other signaling pathways involved in inflammation and fibrosis, such as the NF-κB pathway, further exacerbating the inflammatory response and fibrotic progression in silicosis. Therefore, **CEL-07** can significantly inhibit the progression of silicosis by inhibiting the JAK-STAT and PI3K-AKT pathways [43,44,45].

N-cadherin is involved in the activation and migration of fibroblasts, promoting the formation of fibrotic tissue. Vimentin is an intermediate filament protein that enhances cell motility and contributes to the myofibroblast phenotype. Both N-cadherin and vimentin contribute to the pathogenesis of pulmonary fibrosis by promoting fibroblast activation, migration, and tissue remodeling [46]. In our research, it was demonstrated that **CEL-07** could suppress the expression of N-cadherin and vimentin. Cleaved PARP plays a significant role in pulmonary fibroblasts by inducing apoptosis, preventing excessive proliferation and activation of fibroblasts [47]. This helps regulate silica-induced fibrosis by reducing the accumulation of fibrotic tissue and controlling the progression of fibrosis in the lungs. And it was found that **CEL-07** significantly promoted the expression of cleaved PARP, which might play an important role in controlling the progression of silicosis.

Overall, this study reveals the previously unknown pharmacological properties of **CEL-07** in alleviating both inflammation and fibrosis in the context of silicosis. These findings underscore the potential of **CEL-07** as a promising candidate for the development of drugs against pulmonary fibrosis. Furthermore, the study demonstrates that **CEL-07** alleviates silicosis progression via inducing ROS-mediated apoptosis and regulating the JAK-STAT and PI3K-AKT pathways.

## 4. Materials and Methods

### 4.1. Reagents

Crystalline silica (CS, CAS 7631-86-9) with a mean particle diameter of 1.6 μm and a purity of 99% was acquired from Forsman Scientific (Beijing, China) Co., Ltd. To render the particles endotoxin-free, they were baked at 200 °C for 2 h. Subsequently, the particles were suspended in sterile phosphate-buffered saline (PBS). Before use, the suspensions underwent sonication for 10 min. In-house synthesis yielded Compound **CEL-07**, which had a purity exceeding 95%.

### 4.2. Mouse Experiment

The male C57BL/6 mice, aged 10 weeks and weighing 20 ± 2 g, were obtained from SKBEX Biotechnology (Anyang, China). This animal experiment was conducted in accordance with the Guidelines for the Management and Use of Laboratory Animals and was approved by the Animal Management and Use Committee of Anhui University of Science and Technology (Approval No. HX-001). Prior to the experiment, the mice underwent a one-week acclimation period in a controlled animal facility with regulated temperature and humidity. After acclimation, the mice were randomly divided into three groups: the control group (NC), the model group (CS), and the drug treatment group (CS + **CEL-07**). Each group consisted of 6 mice. Anesthesia was induced using a mixture of isoflurane and air. The mice were then treated with the appropriate doses of CS suspension as follows: NC group received saline (50 μL), CS group received CS (50 mg/mL, 50 μL), and CS + **CEL-07** group received CS (50 mg/mL, 50 μL) for a duration of 28 days, with nasal drops administered every 3 days. The drugs were administered as follows: The NC group received daily intraperitoneal injections of normal saline (100 μL). The CS group received daily intraperitoneal injections of normal saline (100 μL). The CS + **CEL-07** group received daily intraperitoneal injections of **CEL-07** (1 mg/kg, 100 μL) for a duration of 28 days.

### 4.3. Differential Gene Expression in Pulmonary Fibrosis

The microarray data related to rat pulmonary fibrosis were extracted from the Gene Expression Omnibus (GEO) database (https://www.ncbi.nlm.nih.gov/geo/ (accessed on 10 October 2022)) [48]. To identify relevant data, a search for “silicosis” in the GEO database resulted in the selection of the GSE49144 dataset. This dataset comprises gene expression information from 36 rats exposed to CS for 3, 6, and 12 weeks, with 12 rats in each duration group. Differential gene expression analysis between the control group and the CS-exposed group was performed using the GEO2R online analysis tool, which incorporates the GEO query and Limma packages. The selection criteria for differentially expressed genes (DEGs) were set as *p* < 0.05 and log(fold change, FC) > 1 or <−1. The DEGs were then ranked based on the absolute value of log FC, with the top 50 upregulated and downregulated genes chosen for further analysis of differential gene expression [49].

### 4.4. Interaction Network Construction

To perform network analysis of common genes and obtain biological function and pathway insights, we utilized Metascape (http://metascape.org (accessed on 10 October 2022)). This website facilitated gene ontology (GO) and Kyoto Encyclopedia of Genes and Genomes (KEGG) enrichment analysis. To ensure robust results, we set the parameters for overlapping genes and enrichment to 3 and employed a *p*-value cutoff of 0.05. For predicting and constructing protein–protein interaction (PPI) networks, we utilized STRING (https://string-db.org/ (accessed on 12 October 2022)). Additionally, we employed the CytoHubba plugin within Cytoscape 3.8.1 to identify essential nodes in the PPI network. Default parameters for CytoHubba were configured as follows: K-core = 2, degree cutoff = 2, max depth = 100, and node score cutoff = 0.2 [50].

### 4.5. Lung Function Assay (LFA)

Prior to the completion of the experimental phase, the mice from each group were placed in a stable environment for a duration of 30 min to reduce interference. Their lung function was assessed using a whole-body plethysmograph (WBP-4MR). The mice were enclosed in a sealed chamber, and their respiratory function parameters were logged following stabilization by the software (ResMass 1.4.2). The parameters recorded included tidal volume (TV), respiratory rate (RR), minute volume (MV), specific airway resistance (sRaw), forced expiratory volume in 0.1 s (FEV0.1), forced vital capacity (FVC), and peak expiratory flow rate (PEF). The measurements were performed three times, and the outcomes were exported and stored in Excel format.

### 4.6. Histological Staining and Analysis (HSA)

On day 28, mice were subjected to cervical dislocation to ensure euthanasia. Subsequently, the lungs were carefully removed and thoroughly rinsed with saline solution multiple times. The left upper lobe of each lung was immersed in a formalin solution to enable subsequent histological staining methods, including hematoxylin and eosin (HE) as well as Masson trichrome staining. In preparation for immunohistochemical staining, the frozen sections were incubated with the primary antibody overnight at a temperature of 4 °C. Following this, the sections underwent a 50 min incubation at room temperature with the secondary antibody. They were later washed with phosphate-buffered saline (PBS) and stained using DAB. A comprehensive examination and analysis of the stained sections was conducted employing a microscope.

### 4.7. Cell Culture

RAW264.7, BEAS-2B, and NIH-3T3 cells were acquired from the Institute of Biochemistry and Cell Biology, Chinese Academy of Sciences in Shanghai, China. These cell lines were cultivated in a 5% CO_2_ humidified incubator at 37 °C using high-glucose DMEM enriched with 10% fetal bovine serum (FBS) and 1% penicillin/streptomycin. Additionally, the cells were screened for potential mycoplasma contamination and underwent authentication through STR profiling.

### 4.8. Cell Viability Assay

Single-cell suspensions were prepared by resuspending cells in 1 mL of culture medium, followed by seeding them into a 96-well plate with a cellular density of approximately 1 × 10^4^ cells per well. Following 24 h of incubation, the cells were exposed to varying concentrations of **CEL-07** for another 24 h. Cell viability was evaluated using the CCK-8 assay, with measurement of absorbance at 450 nm conducted using a microplate reader.

### 4.9. ROS Generation Assay

An ROS detection kit was employed to assess intracellular ROS levels. DCFH-DA was diluted 1:1000 in serum-free culture medium, reaching a final concentration of 10 μM. The cells were inoculated into a 6-well plate at a density of 2 × 10^5^ cells per well and incubated for 24 h. After being washed with PBS, the cells were treated with **CEL-07** (1 μmol/L or 2 μmol/L) for 6 h. Subsequently, the cells were incubated with DCFH-DA for 30 min in darkness. Fluorescence signals were observed and analyzed using a fluorescence microscope and flow cytometry.

### 4.10. Real-Time Quantitative PCR (RT-qPCR) Analysis

RNA extraction was carried out on cells and lung tissues using Trizol agent. Subsequently, reverse transcription took place utilizing the Revert Aid First Strand cDNA Synthesis Kit. Quantitative amplification of cDNA was carried out using 2× SYBR Green qPCR Master Mix. The primer sequences, designed and verified for specificity using BLAST primer design tool, were acquired from Sangon Biotech. The obtained results were analyzed using the QuantStudio 3 real-time PCR system (refer to Table 1).

### 4.11. Analysis of Cancer Cell Apoptosis

Upon reaching the logarithmic growth phase, the cells were plated onto 6-well plates and subjected to 24 h treatment with **CEL-07** at concentrations of 1 μM and 2 μM. To assess cell apoptosis, the Annexin V-FITC apoptosis detection kit was employed. After staining the cells with Annexin V-FITC and propidium iodide, flow cytometry was utilized for analyzing the population of apoptotic cells.

### 4.12. Western Blot Assay

Cell lysis was performed in RIPA buffer supplemented with protease inhibitors. Subsequently, the proteins were separated using SDS-PAGE and transferred onto PVDF membranes. Following a 1 h blocking step with skim milk, the membranes were incubated overnight at 4 °C with primary antibodies. After washing with TBST, the membranes were incubated with secondary antibodies for 60 min. Visualization and analysis of protein bands were conducted using an imaging system (refer to Table 2).

### 4.13. Data Analysis and Statistics

The data are presented as the mean ± standard deviation (SD) and are derived from a minimum of three independent experiments. Statistical analysis was conducted using GraphPad Prism 8 software. Differences between two groups were evaluated using a two-tailed *t*-test, with statistical significance defined as *p* < 0.05 (* *p* < 0.05, ** *p* < 0.01, *** *p* < 0.001, **** *p* < 0.0001).

## 5. Conclusions

This study uncovers the novel pharmacological properties of **CEL-07** in mitigating inflammation and fibrosis in silicosis, highlighting its potential as a promising candidate drug for pulmonary fibrosis and paving the way for the development of innovative therapeutic strategies for silicosis. It also reveals that **CEL-07** alleviates silicosis progression via inducing ROS-mediated apoptosis and regulating JAK-STAT and PI3K-AKT pathways. However, the molecular mechanism of **CEL-07** remains insufficiently explored, and its direct target is still unclear. In future research, we will employ more advanced technologies and methods to deeply investigate the precise mechanism of action and direct targets of **CEL-07**, thereby providing theoretical support for the further development of **CEL-07** in the treatment of silicosis.

## Figures and Tables

**Table 1:** List of primer sequences for RT-qPCR.

Gene		Sequence 5′----3′
Human-β-actin	Foward	GCACAGAGCCTCGCCTT
	Reverse	CCTTGCACATGCCGGAG
Human-TNF-α	Foward	GAGGCCAAGCCCTGGTATG
	Reverse	CGGGCCGATTGATCTCAGC
Human-TGF-β	Foward	GCTGTATTGCAGACTTAGGACTG
	Reverse	TTTTTGTTCCCACTCTGTGGTT
Human-IL6	Foward	CCCCAATTTCCAATGCTCTCC
	Reverse	CGCACTAGGTTTGCCGAGTA
Human-IL-1α	Foward	AGATGCCTGAGATACCCAAAACC
	Reverse	CCAAGCACACCCAGTAGTCT
Human-IL-1β	Foward	ATGATGGCTTATTACAGTGGCAA
	Reverse	GTCGGAGATTCGTAGCTGGA
Mouse-β-actin	Foward	AGGTCGGTGTGAACGGATTTG
	Reverse	TGTAGACCATGTAGTTGAGGTCA-
Mouse-TNF-α	Foward	CCCTCACACTCAGATCATCTTCT
	Reverse	GCTACGACGTGGGCTACAG
Mouse-TGF-β	Foward	CTCCCGTGGCTTCTAGTGC
	Reverse	GCCTTAGTTTGGACAGGATCTG
Mouse-IL-1α	Foward	GCAACTGTTCCTGAACTCAACT
	Reverse	ATCTTTTGGGGTCCGTCAACT
Mouse-IL-1β	Foward	CGAAGACTACAGTTCTGCCATT
	Reverse	GACGTTTCAGAGGTTCTCAGAG
Mouse-IL-6	Foward	TAGTCCTTCCTACCCCAATTTCC
	Reverse	TTGGTCCTTAGCCACTCCTTC
Mouse-COL1A1	Foward	ACGGCTGCACGAGTCACAC
	Reverse	GGCAGGCGGGAGGTCTT
Mouse-α-SMA	Foward	ACACGGCATCATCACCAACTG
	Reverse	TCCAGAGTCCAGCACAATACCA
Mouse-COL3A1	Foward	CTGTAACATGGAAACTGGGGAAA CCATAGCTGAACTGAAAACCACC
	Reverse	

**Table 2:** List of antibodies for Western blot.

Antibody Name	Item No	Antibody Name	Item No
HRP Goat Anti-Rabbit IgG (H + L)	AS014, Abclonal	Phospho-c-Raf (Ser259)	#9421, CST
Phospho-Jak2 (Tyr1007/1008)	#3771, CST	Phospho-p44/42 MAPK (Erk1/2) (Thr202/Tyr204)	#9101, CST
Phospho-Stat3 (Tyr705)	#9145, CST	N-Cadherin (D4R1H)	#13116, CST
Phospho-PI3 Kinase p85 (Tyr458)	#4228, CST	E-Cadherin (24E10)	#3195, CST
Phospho-Akt (Ser473)	#4060, CST	PARP (46D11)	#9532, CST
Cleaved PARP (Asp214)	#9541, CST	Anti-alpha smooth muscle Actin	#14968, CST
Anti-collagen I Rabbit pAb	GB114197-100, Servicebio	Anti- GAPDH antibody	GB15004-100, Servicebio

## Data Availability

The data presented in this study are available on request from the corresponding author.
